# Metabolomics of infectious diseases in the era of personalized medicine

**DOI:** 10.3389/fmolb.2023.1120376

**Published:** 2023-05-18

**Authors:** Mahbuba Rahman, Herb E. Schellhorn

**Affiliations:** Department of Biology, McMaster University, Hamilton, ON, Canada

**Keywords:** infectious diseases, metabolomics, personalized medicine, biomarkers, multidisciplinary field

## Abstract

Infectious diseases continue to be a major cause of morbidity and mortality worldwide. Diseases cause perturbation of the host’s immune system provoking a response that involves genes, proteins and metabolites. While genes are regulated by epigenetic or other host factors, proteins can undergo post-translational modification to enable/modify function. As a result, it is difficult to correlate the disease phenotype based solely on genetic and proteomic information only. Metabolites, however, can provide direct information on the biochemical activity during diseased state. Therefore, metabolites may, potentially, represent a phenotypic signature of a diseased state. Measuring and assessing metabolites in large scale falls under the omics technology known as “metabolomics”. Comprehensive and/or specific metabolic profiling in biological fluids can be used as biomarkers of disease diagnosis. In addition, metabolomics together with genomics can be used to differentiate patients with differential treatment response and development of host targeted therapy instead of pathogen targeted therapy where pathogens are more prone to mutation and lead to antimicrobial resistance. Thus, metabolomics can be used for patient stratification, personalized drug formulation and disease control and management. Currently, several therapeutics and *in vitro* diagnostics kits have been approved by US Food and Drug Administration (FDA) for personalized treatment and diagnosis of infectious diseases. However, the actual number of therapeutics or diagnostics kits required for tailored treatment is limited as metabolomics and personalized medicine require the involvement of personnel from multidisciplinary fields ranging from technological development, bioscience, bioinformatics, biostatistics, clinicians, and biotechnology companies. Given the significance of metabolomics, in this review, we discussed different aspects of metabolomics particularly potentials of metabolomics as diagnostic biomarkers and use of small molecules for host targeted treatment for infectious diseases, and their scopes and challenges in personalized medicine.

## 1 Introduction

Infectious diseases continue to be the major cause of morbidity and mortality. Disability-related to infectious or communicable diseases have increased over the past several years (WHO) (Boutayeb, 2010; Institute for Health Metrics and Evaluation, 2020; Mundial de Saúde, 2022). The main therapeutic approach for the treatment of infectious diseases are antimicrobial drugs, followed by vaccines as the preventive measures for infectious diseases. However, antimicrobial drug resistance, increased side effects of drug toxicity, and the incidence of vaccine non-responders are increasing (Zimmermann and Curtis, 2019; Szymański et al., 2020; Rahman et al., 2022). To overcome these problems, an in-depth understanding of the hosts’ immune systems and new drug formulations or drug repurposing is essential. While conventional treatment strategy is based on “one-drug fits all”, a tailored treatment strategy for each individual patient can reduce the risks of antimicrobial drug resistance and drug toxicity, mis-use of drug administration, facilitate decisions on drug prescriptions and on the timing of vaccine administration (Cotugno et al., 2019). To facilitate desired changes in the treatment strategy, it is necessary to understand the immune response of the patient in relation to diseased state.

The human immune system consists of two arms as part of the defense mechanism against infection. These are innate immunity and adaptive immunity (Saborano et al., 2019; Purohit et al., 2022). Innate immunity, also known as natural immunity, protects the host from disease-causing organisms by a process known as phagocytosis. Immune cells that are involved in phagocytosis are commonly known as phagocytes and cells that take part in phagocytosis belong to dendritic cells, neutrophils, monocytes or macrophages. In addition to these, basophils, neutrophils, natural killer cells (NK) cells, monocytes, and macrophages also take part in innate immune responses and also activates cells of the adaptive immune system. The adaptive arm of the immune system consists of leukocytes where the majority are T lymphocytes and B lymphocytes. These cells function in the presence of antigen-presenting cells (APCs) and other immune mediators (Saborano et al., 2019; Hartl et al., 2021; Purohit et al., 2022). All cells of the immune system are under the stringent control of the regulatory system. These include genetic, proteomic, and metabolic levels of regulation. Under non-infected conditions, immune cells remain at the dormant condition. Once a pathogen infects the host, these cells become active and respond to the infection through a cascade of signaling molecules that belong to genes, or proteins or metabolites. Immune response also takes place in presence of drugs or vaccines. As a result, the level of metabolites differ in different immune cells or even in non-immune cells (Saborano et al., 2019; Purohit et al., 2022). Metabolites can be detected in different biological fluids such as blood, sweat, urine, plasma, tissues and cells (Saborano et al., 2019; Purohit et al., 2022). In addition to responding to disease states, metabolites also take part in drug metabolism, elimination or inactivation of endogenously or exogenously generated toxic compounds and thereby maintains the homeostasis of cells. Therefore, metabolite profiling can provide an instantaneous readout of the host’s phenotype under different conditions. The study of metabolite profile and metabolic flux is known as metabolomics (Saborano et al., 2019; Purohit et al., 2022).

Metabolomic analyses focus on the quantitative analyses of large numbers of metabolites in biological fluids, mentioned above (Nagana Gowda and Raftery, 2013; Muthubharathi et al., 2021). Metabolites generally have a molecular weight less than 1,000 Da and belong to different chemical groups. Metabolites can be carbohydrates, amino acids or, lipid molecules with overlapping roles in the metabolic pathways of human cells (Purohit et al., 2022). The main metabolic pathways of human cells consists of the glycolytic pathway, pentose phosphate pathway, tricarboxylic acid cycle (TCA), amino acid pathway, beta-oxidation pathway and drug metabolism pathway (Purohit et al., 2022). Due to the complex chemical properties and presence of multiple metabolites in the single sample, technological platforms that exists for the analysis and detection of metabolites ranges from single metabolite detection to multiple metabolite detection platforms and may require cross-validation platforms (Hartl et al., 2021; Castelli et al., 2022). Recently, there has been extensive development on technological platforms, analytical tools and statistical methods to detect metabolites so that metabolomics can be used to understand disease pathogenesis, identify biomarkers that are highly specific to disease, apply metabolites for therapeutic purpose and drug discovery (Adamski and Suhre, 2013; Cronk, 2013). Metabolomics is also used to understand the incidences of treatment non-responders, drug-resistance, drug-relapse and toxicity, vaccine waning and vaccine non-responders. The purpose of research in this area is to help clinicians make decision on patient specific treatment strategy, namely, personalized medicine (Cronk, 2013). Personalized medicine takes into consideration an individual’s genetic profile in response to disease and treatment. This helps to increase and enhance our understanding of pathological conditions more precisely. As indicated by Leroy Hood (Flores et al., 2013), one of the pioneers of this approach, personalized medicine offers deeper understanding of the disease, uses blood as a non-invasive sample for diagnosing and disease identification, segregates complex infections into subtypes of diseases, offers new ways to deal with drug targets, and generates metrics for analyzing health status. In this way, personalized medicine intends to be preventive, predictive, personalized, and participatory and corresponds to the concept of “P4 medicine” (Flores et al., 2013; Beger et al., 2020). Predictive biomarkers (PB) can be used to identify individuals based on how a particular intervention or exposure has affected them, taking environmental and epigenetic factors into account. PB can also be used to predict the likelihood of an upcoming clinical occurrence. Personalization focuses on a patient’s identity determined by their genetic makeup (Flores et al., 2013). Although blood is the major biological fluid used to categorize different biomarkers, other biofluids can also be used to detect metabolites that can be used as biomarkers of disease state (Adamski and Suhre, 2013).

In general, a biomarker is an analyte that can be quantified as a standard indicator of biological processes, pathogenic processes, or response to therapeutic intervention. According to the Food and Drug Administration (FDA), a biomarker must be replicable to the disease state for clinical interpretation. Biomarkers are of several types. These include prognostic biomarker, diagnostic biomarker, monitoring biomarker, predictive biomarker, safety biomarker, pharmacodynamic response biomarker, susceptibility/risk biomarker, and provisional biomarker (Beger et al., 2020). Different biomarkers are used for disease and treatment stratification. In recent years, research on different biomarkers has increased. However, there is little research that integrates diagnostic biomarker information, metabolomics and personalized medicine. This is due to the challenge of combining information from technological platforms, “omics” platforms, which yields larger data sets “big data” and necessitates involvement of experts from different areas including biology, biotechnology, bioinformatics, biostatistics, clinicians, clinical researchers, industries and even regulatory bodies (Beger et al., 2020). Since research leading to biomarker discovery play important role for disease diagnosis, and tailoring of precise medication, omics approaches, particularly metabolomics, offer an intriguing approach for profiling a large panel of molecules in patient samples (Schmidt et al., 2021; Castelli et al., 2022). Additionally, metabolite profiling from biological fluids can be considered as a component of personalized medicine as it enables for the identification of different biomarkers that can be directly linked to a person’s immune response to diseases or drug responses (Purohit et al., 2022). However, the decision to implement a new drug formulation and treatment plan based on biomarker analysis is very expensive and necessitates adequate funding from the health sector. These are variable in different countries and are based on gross domestic product (GDP) (World Health Organisation, 2021). Again, launching of new drugs require approval at different stages such as preclinical testing, clinical tests and post market validation. Despite the challenges, in 2020, 286 personalized medicines were approved by the US Food and Drug administration (FDA) and are on the market. This number accounted for 39 % of the total FDA-approved new drugs and companion diagnostics for both infectious and non-infectious diseases in the year 2020 (Personalized Medicine Coalition, 2020). These drugs and companion diagnostics take into account of the host’s genetic information rather than the organism’s genetic information. This is because host cells are less likely to be mutated compared to the organism’s genetic material. However, small molecules have better potential to be used as biomarkers for disease diagnosis, disease staging, drug toxicity, drug repurposing and drug discovery. Despite the fact that metabolomics reflect the closest phenotype of disease and drug response, no metabolomic tests are included in personalized medicine for diagnostics and targeted drug development for personalized medicine. Most of the diagnostics for infectious diseases are based on molecular technology to detect the pathogen and antimicrobial drugs are mostly inhibitors of enzymes that functions in the synthesis of cell material or nucleic acids of bacteria, viruses, fungi or parasites (Gurevich and Gurevich, 2015). Since small-molecules are closely linked to the host’s phenotype and reflects any dysregulation of metabolic network in response to either the external stimuli or internal diseased state or both (Qiu et al., 2023), in this review, we discussed the potential of small molecules for disease diagnosis and application for targeted therapy, which are ultimate goals for personalized treatment.

## 2 Technological platforms to measure metabolites

Chemical properties of different metabolites of the human metabolic pathway are diverse. Two strategies are used to measure metabolites: targeted approaches and un-targeted approaches. Targeted approaches are used to measure the metabolites that are endogenously synthesized. Metabolites like amino acids, fatty acids, lipids, carbohydrates and bile acids belong to this group. Untargeted approaches, on the other hand, is used to measure metabolites, composition of which is diverse. Untargeted (or unbiased) approaches can provide global metabolite profiles. Untargeted analysis provides information on metabolites that are influenced by the host genome, associated microbiome, and environmental factors. As a result, this approach provides information on metabolites associated with drug dose effects and patient characterization integrating knowledge of genetic variation, metabolism and environmental interventions (Smirnov et al., 2016; Khamis et al., 2019). Since chemical properties of biomarkers are diverse, metabolite detection is largely dependent on analytical chemistry or biochemistry (Ashrafian et al., 2021). However, detection and analysis of metabolites have advanced greatly combining analytical chemistry, biochemistry, biophysics, radiology, nanotechnology, artificial intelligence, bioinformatics and electrophysiology (Ashrafian et al., 2021). It is beyond the scope of this review to discuss all of these technological platforms. Therefore, we will focus on the technologies that are used to detect metabolic changes in mammalian cells upon microbial infection.

Based on the intrinsic operating principle, metabolite detection technologies are categorized into biochemical assays, cell-based assays and biophysical methods. The biochemical assays use cell-free *in vitro* techniques to detect the biochemical reactions that occur in a subset of cellular processes. Cell-based assays, on the other hand, use live cells as model to assess the biochemical changes that occur in healthy or diseased cells (Cronk, 2013). Table 1 shows some examples of biochemical and cell-based assays.

**TABLE 1:** Selective technological methods for metabolite detection other than NMR and MS.

Type of assay	Technology	Detection method	Description	References
Biochemical assay	Ligand binding assays	Radioactive assay using Isotopes such as ^3^H or^125^I are typically used	Enzymatic assays, protein-protein interactions, receptor-ligand interactions	Arkin et al., 2004; Cronk (2013), Ahl et al. (2020)
	Fluorescence Technologies	Fluorescence intensity	Enzymatic assay using a fluorogenic assay	
			Enzymatic analyses using fluorescence quench assays	
		Fluorescence resonance energy transfer (FRET)	Enzymatic assay	
		Time resolved fluorescence (TRF)	Measurements of the second messengers cAMP and inositol triphosphate (InsP3), immunoassays, analysis of kinase enzyme activity, monitor protease enzyme activity, cytokine measurement	
		Fluorescence correlation methods (FCM)	Single molecule detection	
Cell-based assays	Fluorometric assays	Fluorometric assays-uses a range of fluorescence dyes	Detect alterations in intracellular concentration of cAMP	
	Fluorescence technologies	Fluorescence resonance energy transfer (FRET)	Enzymatic assay	
		Flow cytometry	Analysis of the presence of cell surface proteins and/or metabolites	
	Label free detection platforms	Phenotype Microarray-multiplexed	Detection of cellular energy level in the presence of various substrates and monitoring kinetics of substrate utilization of particular metabolic pathway	

Biophysical methods investigate the structure, properties and dynamics or function of biomolecules at the atomic or molecular level (Cronk, 2013). We discussed this method in the next section.

## 3 Metabolomic workflow for personalized medicine

**FIGURE 1:**
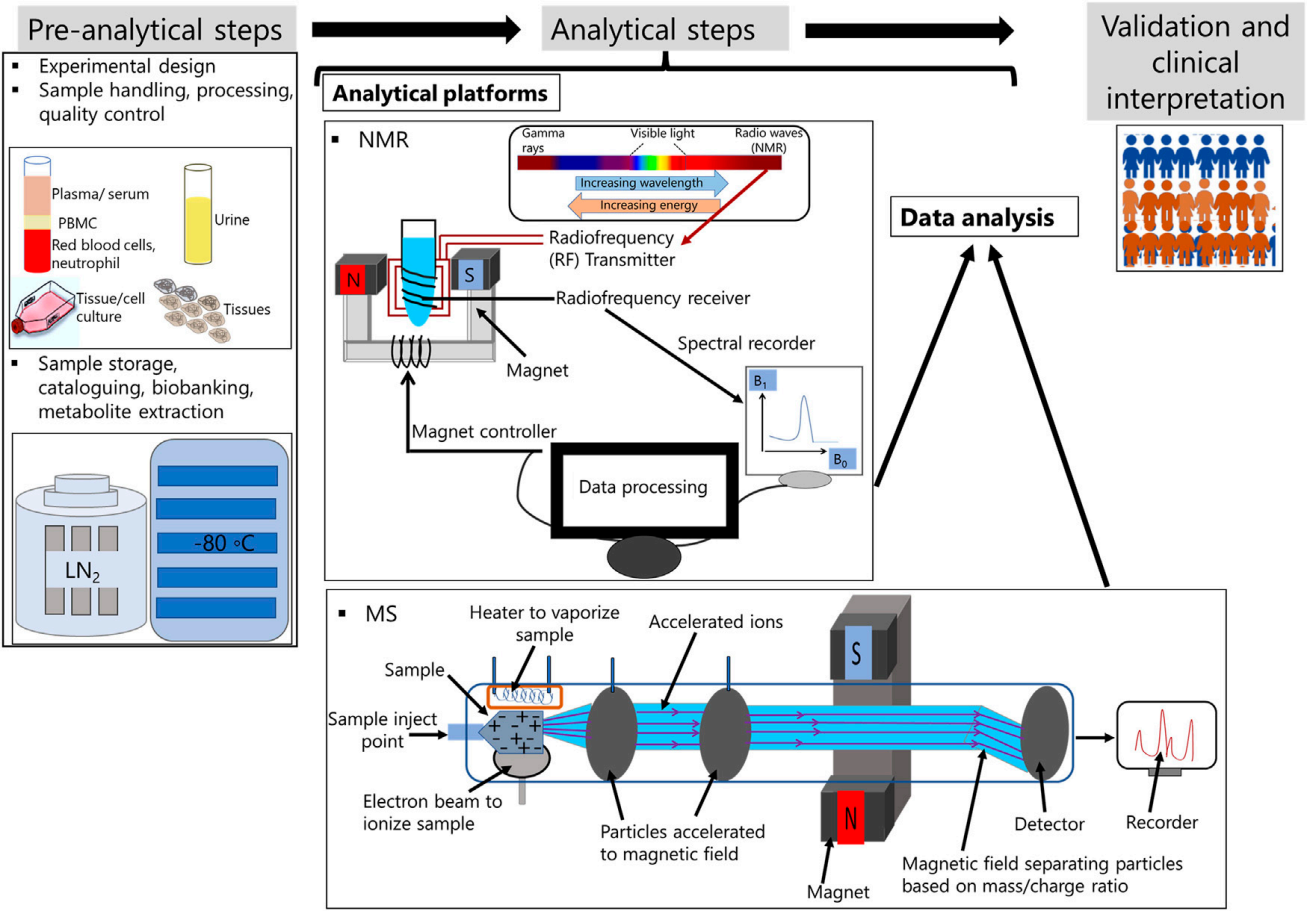
Metabolomic workflow for personalized medicine.

Personalized medicine is an emerging field of treatment strategy, where metabolomics is regarded as a novel approach methodology (NAM). Thus, technological platforms that can be used to detect all metabolites present in the sample require a well-organized workflow to reduce technical artefacts and implement data for the identification of patient and disease specific biomarkers in clinics. Incorporation of metabolomics for personalized medicine workflow involves three basic steps. These are 1) pre-analytical steps, 2) analyte detection and 3) validation and clinical translation (Figure 1) (Nordström and Lewensohn, 2010; Trivedi and Goodacre, 2020).

### 3.1 Preanalytical step

This step involves well-organized experimental design on selection of sample type, quenching solution, technological platforms for metabolite extraction and separation, and selection on the type of analytical software to be used for metabolite detection. Factors that needs to be considered with importance at this steps are type of containers for sample collection and storage, sample handling, transport and extraction (Di Minno et al., 2021). Recently, biobanking has become an integral part of personalized medicine. Biobanks are operated under ISO 13485 (Müller et al., 2020; Dagher, 2022). and these are context based., i.e., biological samples can be collected from healthy donors or these can be individuals affected by disease. Samples are either collected manually or by automated liquid handlers. The latter reduces batch to batch variations (MacDonald, 2020). Research labs that are involved in epidemiological studies can use standardized protocols for sample collection, storage and donor’s information on whole-genome, genotype, geographic location, dietary preference, proteome and medical images. These information are leveraged from national registries and also incorporated in electronic health records (EHRs) (Hewitt, 2011; Kirwan et al., 2018). Biobanking for both communicable and non-communicable diseases is available. For infectious diseases or communicable diseases, biobanking for tuberculosis, Zika virus, dengue, and, the most recent novel coronavirus, severe acute respiratory syndrome coronavirus 2 (SARS-CoV-2) are available (Sgaier et al., 2007; Ramanathan et al., 2020). Biobanks for infectious diseases facilitates rapid development and deployment of diagnostics, for example, point of care testing (POCT) (Ramanathan et al., 2020).

### 3.2 Analytical methodology

This encompasses biophysical methods that enable atomic- or molecular-scale investigation into the structure, characteristics, dynamics, or function of biomolecules. X-ray crystallography-based assays, chromatography, nuclear magnetic resonance spectroscopy (NMR), mass spectrometry (MS), and surface plasmon resonance SPR) are examples of common biophysical based methods. Of these, MS and NMR are utilized in both research labs and medical facilities (Frédérich et al., 2016; Di Minno et al., 2021).

NMR was initially developed to identify protein nuclei molecules. Nuclei within the same molecule can absorb energy at different frequencies. The chemical milieu of the nuclei has a direct impact on these chemical transformations. The emission and absorption of electromagnetic radiation can be observed if nuclei are placed in a strong external magnetic field that generates vibrations. Molecular vibration modes can be recorded as radio frequency (RF) just below visible red light. This RF is transformed into spectra in NMR (Frédérich et al., 2016; Di Minno et al., 2021). NMR is the most advantageous technique for metabolomics research due to its high reproducibility, simple sample preparation, and the ability to measure a broad range of small molecule metabolites. In diagnostic labs, NMR is used because sample collection is relatively non-invasive, simple, reproducible, low cost per sample preparation, and easy to handle large datasets. There are also bench-top NMR platforms available for clinical diagnostics laboratories. NMR IVDr is used in pre-clinical screening research laboratories to monitor the effect of infectious disease such as SARS-COV-2 infection on multiple organ systems (Sample et al., 2023). There are different types of NMR-based quantitative metabolomics platforms available. These include identification and quantification of metabolites via localized *in vivo* NMR, lipid and lipoprotein identification, and small molecule identification. NMR spectrometry is one-dimensional (1D) and two-dimensional (2D). 2D NMR are effective for reducing spectral complexity and identifying metabolites in complex samples. Although major advantage of NMR technology is that data are reproducible, drawbacks of the technology is low sensitivity compared to MS (Di Minno et al., 2021) (Frédérich et al., 2016).

The use of MS as a biomedical diagnostic technology has increased over the past several years. The principle of mass spectrometry is that a charged particle is passed through a magnetic field and diverts it along a circular path with a radius proportionate to the mass to charge ratio, m/e (Frédérich et al., 2016; Di Minno et al., 2021). Specific types of mass analyzers are used in metabolomics. MS methods coupled with previous separation modalities such as gas chromatography (GC), liquid chromatography (LC), and capillary electrophoresis (CE), provide information on the chemical properties of the metabolites, and thereby, are useful for metabolomics studies. At present, direct flow infusion mass spectrometry (DIMS) and capillary electrophoresis mass spectrometry (CE-MS) are used in clinics. The advantages of MS over NMR technology are its high selectivity and sensitivity. Metabolites at the femto level concentration can be measured using MS. However, major drawbacks are the long time required for the analysis of the sample and batch to batch variation (Frédérich et al., 2016; Di Minno et al., 2021).

Of the NMR and MS platforms, NMR is commonly used for untargeted approach. Untargeted approach (global profiling) is an important tool for personalized medicine (Castelli et al., 2022).

While NMR and MS platforms are used to identify or measure metabolites, these platforms are also used to analyze samples and determine the metabolic flux, which is a measure of the rate at which metabolic reactions occur. Traditional metabolite quantification does not provide intracellular metabolic rates or associated pathway activity. Metabolic fluxes (fluxomics) provide more precise data on the metabolic activities of specific pathways. The fluxome is constrained by the cell’s metabolic profile and corresponding stoichiometry. Each metabolic reaction’s rate is also influenced by a number of upstream factors, including gene expression and regulation, enzyme concentration, enzyme phosphorylation status, and metabolite concentrations related to the reaction. Therefore, metabolic flux measurements provide a complete and dynamic depiction of the metabolic state of the cell by capturing the net interplay of the transcriptome, proteome, regulome, and metabolome. Metabolic fluxes are of two different categories. Extracellular flux and intracellular flux. The extracellular flux crosses the cell membrane and shows the impacts of the medium, such as glucose uptake, or cell growth and biomass. By monitoring the changes in extracellular metabolite concentrations or biomass over time, extracellular fluxes can be measured directly. Intracellular flux, which does not cross the cell membrane, cannot be measured directly. Their impact can be observed by measuring the frequency of integration of an isotopically labelled substrate such as ^13^C-glucose into a biological system of organisms, cells or animals. Due to the metabolic reactions, the labelled carbon sources are converted into intermediate metabolites and secreted products. The metabolite’s overall isotopic composition patterns are influenced by the reaction fluxes and carbon atom rearrangements. In addition to ^13^C tracers, ^15^N and ^2^H labelled isotopes are also used in fluxomic studies. Flux is calculated based on the experimental external fluxes, mass distribution vectors (MDV) and a metabolic map that highlights all relevant reactions. Software such as, eiFlux, INCA, METRAN, OpenMedius and 13C2FLUX are used to support the metabolic model, flux estimation, and confidence interval calculation (Moiz et al., 2022). Metabolomic flux analysis has been deployed to investigate the effect of the drug Bedaquiline (BDQ) in antibiotic resistant strain of *Mycobacterium tuberculosis* (Mtb). MFA has also been used to study the metabolic changes in host cell during viral infection (Moiz et al., 2022).

### 3.3 Validation and clinical translation

Validation of metabolite based biomarkers aims for reproducibility across several cohorts and requires standardization. The concentration of metabolites and type of metabolites varies depending on the sample type. Therefore, metabolites to be used for clinical interpretation or used as biomarker require approval from regulatory bodies to interact this with the disease stage or treatment response. Biobanking of patient samples and longitudinal observation provide reliable data on a robust biomarker. However, the number of controlled studies specially examining infectious diseases and biobanking are very few (Ramanathan et al., 2020). Furthermore, metabolomics alone cannot be used to choose robust biomarkers unless other omics technologies are integrated (Rhee, 2020). Only a small number of research have combined genotyping and metabolomics data for genome-wide association (mGWAS) studies testing different ethnic group and demographic regions (Adamski and Suhre, 2013; Trivedi and Goodacre, 2020). Future studies require inclusion of more studies for validation of disease cohort and discovery cohort for personalized medicine (Yet et al., 2016).

## 4 Metabolic biomarkers and therapeutics for infectious diseases

According to World health Organization (WHO), infectious diseases cause disability even after post-infection sequel (https://www.who.int/data/gho/data/themes/mortality-and-global-health-estimates). Table 2 shows disability-adjusted life years (DALYs) from several infectious diseases. In this section we discuss some of the infectious diseases shown in Table 2 with higher prevalence and DALYs, how these pathogens modulate the host’s metabolic pathways and potential of metabolites to be used as biomarkers of disease diagnosis and drugs and diagnostics available approved by FDA for personalized treatment of infectious diseases and antimicrobial drugs targeting the host metabolic pathways.

**TABLE 2:** Global prevalence, incidence, death, DALYs, YLDs and YLLs of selected communicable diseases in 2019. The numbers are calculated per million and shows age-standardized rates to reflect the global burden of disease (GBD).

Disease category	Type of disease	Prevalence (in millions)	Incidence (in millions)	Deaths (in millions)	YLLs	YLDs	DALYs
Communicable diseases (CD)	Total (CD)	4,540	26,400	10·2	564	104	669
	Human immunodeficiency virus (HIV)	36·8	1·99	0·864	43·6	4·01	47·6
	Acute hepatitis C	0·636	5·51	0·00548	0·244	0·00891	0·253
	Acute hepatitis B	9.23	80	0.0325	1.46	0.159	1.61
	Upper respiratory infections (Influenza A and others)	237	17,200	0.00946	0.499	5.89	6.39
	Dengue	3·39	56·9	0·0361	1·83	0·552	2·38
	Tuberculosis	1830	8·50	1·18	42·7	4·32	47·0
	Malaria	181	231	0·643	43·8	2·61	46·4
	Leprosy	0·528	0·0527	−	−	0·0288	0·0288

Note: YLLs, years of life lost; YLDs, years lived with disability; DALYs, disability-adjusted life years.

[Data source: https://www.healthdata.org/results/gbd_summaries/2019/ World Health Organization (WHO)].

### 4.1 Metabolomics of host cells in response to infection

#### 4.1.1 Virus infection and host metabolism

##### 4.1.1.1 HIV/AIDS

The human immunodeficiency virus (HIV) causes symptoms resembling the flu (influenza) and if left untreated, can lead to acquired immunodeficiency syndrome (AIDS). The virus damages the immune system in such a way that the patient becomes more vulnerable to opportunistic infections, mostly by tuberculosis (Personalized Medicine Coalition, 2021). Table 2 shows the incidence, prevalence, YLLS, YLDs and DALYs. The majority of the disease burden is carried in the sub-Saharan Africa super-region accounting for 64.9% of new HIV infections and 74% of all deaths caused by the virus worldwide. Despite the absence of a treatment, HIV patients can experience chronic infection while on antiretroviral therapy (ART). Due to the enormous improvements in HIV detection and treatment, this is now possible to implement (Tounta et al., 2021).

Human immunodeficiency virus type 1 (HIV-1) and type 2 (HIV-2) both contribute to the development of AIDS. Although HIV-2 exhibits lower virulence and transmission than HIV-1, and only 30% of HIV-2 infections progress to AIDS, HIV-2 is nevertheless responsible for the majority of cases of AIDS worldwide. Before symptoms appear and the disease worsens, there is an asymptomatic stage that might continue years after the acute and original infection. As the virus adheres to and infects the cells to multiply within them, infection causes a progressive decline in CD4^+^ T-cells even during the asymptomatic phase. Compared to HIV-1, HIV-2 is less contagious and has a slower rate of CD4^+^ T-cell depletion (Tounta et al., 2021).

In HIV research, metabolomics has proven to be a useful technique for both diagnosis and vaccine development. Studies investigated indicators in biofluids like plasma that correlate with the level of protection provided by potential vaccinations. Initial research determined that comparing the metabolic patterns of patients’ serum renders it possible to distinguish between HIV^+^ and HIV^−^. The distinction between HIV^+^ patients who had received antiretroviral medication (ART^+^) and HIV-positive patients, based on considerable alterations in glucose and lipid levels, was a more intriguing conclusion. Untargeted ultrahigh-performance liquid chromatography UHLC/MS/MS and GC/MS of plasma and cerebrospinal fluid (CSF) were used by Cassol et al. to validate these findings. The changes found in HIV^+^ ART^+^ samples involved neurotransmitters (glutamate, N-acetylaspartate), myo-inositol, and ketone bodies, and their prevalence suggested an effect similar to increased aging. The results provided understanding of the inflammatory and neurotoxic processes at play because the discovered metabolites were also among the top classifiers for the emergence of HIV-associated neurocognitive disorders (HAND) (Diray-Arce et al., 2020; Tounta et al., 2021).

The metabolic profiles of HIV-1 and HIV-2 infections, as determined by LC/MS, were compared in order to identify the factor contributing to the decreased pathogenicity of HIV-2. Even though the profiles for glycolysis and TCA were identical, the HIV-2 profile was distinguished by a rise in deoxynucleotide triphosphates (dNTPs), which are thought to be related to HIV-2 viral protein x (Vpx). SAMHD1, a host antiviral protein with dNTPase activity that works to reduce the availability of dNTP for viral reverse transcription, has been linked to Vpx’s role in degrading SAMHD1. LC-MS/MS metabolites were extracted from both HIV-uninfected and HIV-infected primary monocyte-derived macrophages. The HIV-1 strains were associated with increases in glyceraldehyde 3-phosphate (G3P) and fructose 1,6-bisphosphate (FBP), but the rise in quinolinate was the most notable modification. Quinolinate is an intermediate in the kynurenine pathway, which begins with the breakdown of tryptophan, which produces NAD^+^. NAD^+^ was not noticeably reduced despite the alterations in quinolinate levels that were seen. The kynurenine pathway’s impaired function has been linked to a number of conditions, including neurodegenerative illnesses and chronic inflammation. The immunological response has also been linked to tryptophan levels, and ongoing tryptophan depletion has been associated to T cell exhaustion and tryptophan catabolism towards immune activation. These findings raised the possibility that tryptophan levels may be responsible for the distinction between HIV-1 and HIV-2 pathogenicity (Diray-Arce et al., 2020; Tounta et al., 2021).

Biofluids such urine, whole blood, and serum have been used in metabolomic investigations to find metabolite markers associated with HIV-induced oxidative stress (OS). Studies have investigated changes suggestive of OS, such as altered amino acid metabolism, including those of alanine and glutamine, using a variety of techniques (NMR, LC/MS, GC/MS, UPLC/MS). Using a DB-5 MS capillary column and 105 plasma samples from HIV^+^ sub-Saharan people, Bipath et al. carried out GC/MS analysis. They discovered elevated levels of indoleamine 2,3-dioxygenase (IDO) in the HIV^+^ samples. Using a DB-5 MS capillary column and 105 plasma samples from HIV^+^ sub-Saharan people, Bipath et al. carried out GC/MS analysis. They discovered elevated levels of indoleamine 2,3-dioxygenase (IDO) in the HIV^+^ samples. In contrast to the findings from HIV^−^ and HIV^+^ samples from higher-income countries, this rise led to an accelerated breakdown of tryptophan and a buildup of kynurenine pathway intermediates such as quinolinate and compounds with neurotoxic characteristics (Diray-Arce et al., 2020; Tounta et al., 2021).

Table 3 shows some of the metabolites that have been detected in biological samples of HIV patients.

**TABLE 3:** Metabolites detected in patient’s samples as a result of microbial infection.

Disease	Species	Type of infection	Sample	Technique	Metabolites/pathways	References
Virus	*Lentivirus*	Human immunodeficiency virus/HIV	CSF, plasma	^1^H-NMR, LC–MS, targeted LC–MS	acetate, citrate, creatine, dicarboxylicacylcarnitines, dopamine, glucose, glycerophospholipids, glycolysis, L-aspartate plasmalogen/plasminogen, lysophospholipids, methylglutarylcarnitine, phosphatidylcholines, sphingomyelin, sphingosine-1-phosphate	Diray-Arce et al. (2020)
	*Flavivirus* (DENV) (Dengue virus)	Dengue	Serum	GC-MS, LC-MS	acylcarnitines, amino acids, bile acids, chenodeoxyglycocholic acid, galactose and pyrimidine, glycine, glyoxylate and dicarboxylate, kynurenine, pentose phosphate pathway, phospholipids, propanoate, purines, serine, serotonin, starch and sucrose, threonine, uric acid, fatty acid synthesis, glycolysis	Diray-Arce et al. (2020)
	*Alphainfluenza virus*	Influenza	Plasma	*1*H-NMR, GC–MS	amino acids and ketone bodies, cAMP, glucose, glutathione, lipid, N-acetylglucosamine(O-GlcNAc), purine	Diray-Arce et al. (2020), Tounta et al. (2021)
				^1^H-NMR,	Citrate; Fumarate; 3-Methyl,2-Isovalerate; Alanine; Tyrosine; Methionine; Histidine; 4-Hydroxybutyrate	Banoei et al. (2017)
				GC–MS	Uric acid, Tyrosine, Citric acid, Asparagine, Myoinositol, Lysine, Arabinonic acid Threonine, Aspartic acid, Threonic acid	
	*SARS-CoV-2*	COVID-19	Plasma, serum	LC-MS	Bile acids, bilirubin, diacylglycerols, free fatty acid, glucose, glucuronate, glycerol 3-phosphate, kynurenine, lysophosphotidylcholines, malic acid, monosialodihexosylganglioside, phosphatidylcholines, sphingomyelin, triglycerides, tryptophan	Diray-Arce et al. (2020)
			Serum	LC-MS	L-phenylalanine, tyrosine	Qiu et al. (2023)
			Serum	GC-MS	Pyruvate, lactate, succinate, a-ketoglutarate	
	Hepatitis B virus (HBV)	Infection	Serum	GC/TOF	Asparagine, β-glutamate, glycerol, and glucose.	Tounta et al. (2021)
				UPLC/MS	Ornithine, citrulline and glutamate	
	Hepatitis C virus (HCV)	Infection	Plasma	^1^H-NMR, LC-MS, GC-MS	Induces activation of SREBPs and FASN to induce fatty acid synthesis, phosphatidylcholine, phosphatidylethanolamine, cholesterol, sphingolipids, glycolysis	Tounta et al. (2021)
Bacterial	*Escherichia coli*	Urinary tract infection	Urine	^1^H-NMR, LC-MS	acetate, amines, aspartic acid, cadaverine, citrate, glutamic acid, glycine, hippurate, trimethylamine, trimethylamine n-oxide	Tounta et al. (2021)
	*Mycobacterium tuberculosis*	Tuberculosis	Plasma, Serum	LC-MS, FIA-MS, GC-MS	amino-acyl tRNA, asparagine, aspartate, citrulline, cysteine, gamma-glutamylglutamine, fatty acid metabolism, glutamate, glutamine, histidine, inosine, kynurenine, lysophosphatidylcholines, medium chain fatty acid, lysosome pathway, mannose methionine, protein digestion pathway, sphingolipid, sphingosine-1-phosphate, sulfoxymethionine, tryptophan, urea	Tounta et al. (2021)
Parasites	*Plasmodium falciparum and Plasmodium vivax*	Malaria	Plasma, Urine	^1^H-NMR, UHPLC-HRMS	LDL/VDL, lactic acid, isoleucine, glycoprotein, pipecolic acid, taurine, N-acetylspermidine, N-acetylputrescine, 1,3-diacetylpropane, tyrosine, glucose, alanine, creatine/phosphocreatine, N-acetylglutamate, salicylurate, N-acetylornithine, valeylglycine, pipecolic acid, biopterin-3-hydroxybutyrate	Tounta et al. (2021)

##### 4.1.1.2 Hepatitis B

About 350 million infections and 600,000 deaths occur worldwide each year, primarily in Asia and Africa, due to the blood-borne hepatitis B virus (HBV). It can cause acute infection to chronic hepatitis B leading to LF, LC, and HCC. Metabolomics can have a significant influence on hepatitis B virus (HBV) research by offering a sensitive approach to determine the disease stage without the requirement for potentially harmful tests like biopsies and histopathology. The next stages are liver fibrosis (LF) and cirrhosis, which can advance to hepatocellular carcinoma (HCC), once an acute infection transforms into a chronic condition. Researchers used metabolomics platforms such as GC/TOF to identify metabolic biomarkers in serum samples to distinguish between HBV stages and enable the early detection of HCC. Asparagine and glutamate were found to be associated with HBV infection, cirrhosis progression, and changes to distinguish cirrhosis from HCC. For the development of HCC, major alterations of metabolites of the key pathways like glycolysis and the TCA cycle were reported. A blockage of the TCA cycle was reported with increases in malic acid, citric acid, and succinic acid, and a dependency on glycolysis. Additionally, palmitic acid for the detection of cirrhosis against HBV and phenylalanine, malic acid, and 5-methoxytryptamine was proposed as possible biomarkers for discriminating between HBV and controls. In order to examine the metabolome during chronic HBV and for disease staging, UPLC/MS was used to detect metabolites from serum samples. An elevated ornithine levels together with elevated citrulline and glutamate levels, which indicated dysregulation of the urea cycle could be linked to liver injury. Additionally, the virus also used the glycerol-3-phosphate NADH shuttle as a vehicle for its reproduction (Tounta et al., 2021).

##### 4.1.1.3 Hepatitis C

According to the WHO (2018), 70% of individuals who contract the hepatitis C virus (HCV) proceed to develop a chronic illness. Hepatitis C (HCV) is caused by a flavivirus (Paula et al., 2012). While the acute phase typically only causes minor symptoms, Hepatocellular carcinoma (HCC) and cirrhosis are possible outcomes of chronic HCV infection resulting in liver fibrosis (LF), which develops over time. According to the METAVIR scale, the stages of fibrosis are frequently categorized as F0, F1-2, F3, and F4. These stages range from cirrhosis to no symptoms of fibrosis, and one of the greatest risks of the latter stages is further development into HCC (Tounta et al., 2021).

Enzyme immunoassays (EIA) for the detection of anti-HCV and Polymerase chain reactions (PCR) for the identification of HCV-RNA are the standard techniques used in the screening and diagnosis of HCV-infections. These methods involve a lot of time and come with a risk of contamination, and the increased expense of the procedure prevents them from being used on a regular basis. Although liver biopsy is presently regarded as the gold standard method for staging the HCV-associated LF, it has several disadvantages, such as the possibility of bleeding, acute discomfort, and organ perforation. As a result, scientists are concentrating on developing alternative approaches that are easy to use, cheap to produce, safe to use, and quick to screen for HCV infection. Due to the significant incidence and fatal outcomes of the HCV-induced HCC worldwide, metabolomics studies have been used for early diagnosis and understanding of the correlation of metabolites with the progression of the disease (Tounta et al., 2021).

Utilizing NMR, biomarkers for disease detection have been discovered, and by combining MS with other chromatographic techniques, changes in sugar metabolism and elevated levels of metabolites, such as glucose, in the blood, were observed. Similar to HBV, metabolomics can offer a precise and non-invasive tool for disease staging, and numerous studies in this area have been carried out. Data mining and multivariate statistical analysis were used to create one of these algorithms, which attempted to use the methods of statistical analysis to create an algorithm to distinguish between stages by using amino acid ratios in plasma based on the formula [(phenylalanine)/(valine) + (threonine + methionine + ornithine)/(proline + glycine)]. Biopsies were used to assess the extent of fibrosis in 53 individuals’ plasma samples. The formula successfully distinguished between F3-F4 and earlier phases, according to the results, and successfully distinguished F4 from all other stages. The area under the receiver operator curve was used to assess performance, and the findings showed good confidence (95% confidence interval). Although these findings are preliminary, they could be used to assess liver fibrosis without invasive procedures. Another study used LC/MS and GC/MS to find changes that may be used as fibrosis markers, such as cysteine and bile acids (Tounta et al., 2021).

NMR has also shown promise in the staging of diseases. A ^1^H-NMR technique was used to detect NMR spectra of serum samples from 67 patients with HCV, 50 with HBV, and 43 healthy controls in order to characterize their metabolic fingerprints (Tounta et al., 2021).

Increases in the metabolites such as lactate, 3-hydroxybutyrate, acetate, and pyruvate after HCV infection suggest activation of the glycolysis pathway, which the virus is thought to have triggered. The use of metabolomics in HCV diagnosis and staging can significantly benefit from the findings for HBV (Tounta et al., 2021).

##### 4.1.1.4 Dengue

Dengue virus belongs to the genus of flaviviruses, which also includes yellow fever virus, West Nile virus, tick-borne encephalitis virus, and Zika virus; all are arthropod-transmitted infections. Four different serotypes of dengue virus exist. The virus DENV can cause clinical illness such as dengue fever, dengue hemorrhagic fever, dengue shock syndrome, chronic fatigue syndrome or death. In 2019, dengue resulted in 2.38 million DALYs and 36,100 deaths. Among all neglected tropical diseases in South-east Asia in 2019, dengue had the largest DALY burden (Diray-Arce et al., 2020). Infected host cells exhibit increased fatty acid synthesis and glycolysis (Table 3) (Personalized Medicine Coalition, 2021). There is no specific treatment or vaccine for dengue.

There are four distinct serotypes of the DENV that cause dengue fever (DF), designated as DENV-1 through DENV-4. A minimum of 5% of DENV-mediated DF develops into severe forms, such as dengue hemorrhagic fever (DHF) and dengue shock syndrome (DSS), which can be fatal. Despite the fact that the majority of DENV-mediated DF is asymptomatic or only manifests mild symptoms (fever, rash, and joint pain). One serotype’s infection does not offer defense against another. Therefore, accurate diagnosis and prognosis depend on early and precise disease identification. Metabolomics is crucial for achieving this goal. Several studies that compared the metabolomes of DENV-infected and non-DENV-infected patients found metabolites that might be utilized to diagnose DENV and predict the prognosis of severe to mild DENV disease. These research articles describe how sphingolipids and glycerophospholipids are affected by DENV infection. Sera from DENV-infected patients exhibit lower amounts of phosphatidylcholine (PC), lysophosphatidylcholines (LPC), and lysophosphatidylethanolamines (LPE), as well as decreased levels of sphingomyelin (SM) in contrast to healthy control participants. The LPE correlates with the host’s later response while the increased SM correlates with the host’s early response. These investigations thereby indicate the potential of these lipids as diagnostic and prognostic biomarkers (Diray-Arce et al., 2020; Tounta et al., 2021).

##### 4.1.1.5 Influenza

Seasonal influenza is an acute respiratory infection which is caused by four types of seasonal influenza viruses. These are influenza virus A, B, C, and D. WHO estimates that seasonal Influenza may result in 290,000 to 650, 000 death each year due to respiratory diseases alone (Lajoie, 2015). Clinical symptoms of seasonal influenza include sudden onset of fever, dry cough, headache, muscle and joint pain, sore throat, runny nose and severe malaise. Although most people recover from the symptoms within a week, influenza can cause severe illness or even death in patients with chronic medical conditions such as chronic cardiac, pulmonary, renal, metabolic, liver or hematological diseases, immunosuppressive conditions such as HIV/AIDS or patients receiving chemotherapy or suffering from malignancy (Karlsson et al., 2021).

The management of the disease can be greatly improved by the early use of antiviral medications in patients who have been diagnosed with H1N1 influenza pneumonia. Increased ICU admission and mortality have been linked to delaying treatment for pneumonia caused by the H1N1 virus. With the use of biomarkers, viral pneumonia may be diagnosed and prognosed earlier, treated more effectively, and new knowledge about pathophysiologic processes may be gained. Applying metabolomic profiling is one promising strategy for discovering disease-related biomarkers. Through the use of nontargeted ^1^H-NMR and GC-MS methods, researchers were able to distinguish H1N1 pneumonia from bacterial CAP (community acquired pneumonia) and ventilated ICU control subjects through metabolomic profiling of plasma samples collected within 24 h of hospital admission. Additionally, by separating H1N1 non-survivors from survivors using samples taken from inside the study population, researchers further hypothesized that plasma metabolomics may be employed for the prognosis of mortality (Banoei et al., 2017). Metabolites detected in samples of influenza patients include amino acid pathway, glycolysis, lipid and purine metabolism pathway (Table 3).

##### 4.1.1.6 SARS-CoV-2

The virus, severe acute respiratory syndrome coronavirus 2 (SARS-CoV-2) is a strain of coronavirus family and causes the disease COVID-19. The outbreak caused by SARS-CoV-2 caused over 100 million confirmed cases in December 2019, and almost 2.5 million fatalities by 2021. Since there is no effective treatment, an immediate and accurate diagnosis is required. Metabolomics is an effective technique for combating COVID-19 because of its speed and ease of use. This method produces large amounts of information and enables the quick screening of molecules for the identification of biomarkers for the diagnosis and prediction of disease severity. With the use of LC/MS and NMR, several researchers found inflammation associated metabolites such as increased level of alpha-1-cis glycoprotein. There was an increased ratio of kynurenine/tryptophan in COVID-19 patients with diabetes and metabolic disorders. Changes in arginine and kynurenine ratio in plasma samples of COVID-19 patients were also detected by LC/MS/MS and ^1^H-NMR. Alterations in amino acid and carbohydrates were found to be linked to disease severity in COVID-19 patients (Table 3) (Tounta et al., 2021).

#### 4.1.2 Bacterial infection and host metabolism

##### 4.1.2.1 Tuberculosis (TB)

*Mycobacterium tuberculosis* is the etiological agent of tuberculosis. It is a contagious disease. Both pulmonary and extrapulmonary TB are caused by the bacteria. The bacteria cause both drug-susceptible and drug-resistant TB. In 2019, there were 1.8 million TB-related deaths and 8.5 million new cases of TB (Table 2) (Institute for Health Metrics and Evaluation, 2022). The pathogen is a facultative intracellular pathogen. The main target organ for *M. tuberculosis* colonization is the lung, which results in pulmonary tuberculosis (TB). *M. tuberculosis* predominantly adapts to intracellular habitats in macrophages. In a specialized phagosomal compartment within these host cells, *M. tuberculosis* survives and reproduces. The bacteria appear to be capable of escaping into the cytoplasm of the host cells under specific circumstances. Immediately upon infection, granulomas develop, which contain an infected macrophage core encircled by foamy macrophages, monocytes, and multinucleated giant cells. *M. tuberculosis* may also infect other organs in immune-compromised individuals, leading to extrapulmonary TB. It has been suggested that an essential reservoir for the persistence of *M. tuberculosis* is the visceral adipose tissue (Tounta et al., 2021).

A lot of work has been done on understanding *M. tuberculosis*' complicated metabolism both *in vitro* and *in vivo* due to its importance to healthcare. The metabolic reactions of the target host cells and tissues to *M. tuberculosis* infections are much less understood, hence this issue is far from being fully resolved. Several investigations involving infected host cells (mostly macrophages) and animals involved transcript profiling. Additionally, *M. tuberculosis*-infected cells and animal models were subjected to proteomics and metabolomics analyses. These investigations gave researchers important insights into the nature of the immunological responses that *M. tuberculosis* causes, as well as some hints about how it affects the host cells' metabolic processes (Tounta et al., 2021).

The majority of the metabolic alterations seen in host cells infected with *M. tuberculosis* are connected to defense mechanisms, such as oxidative stress and the synthesis of antimicrobial peptides. By producing RNI from iNOS, macrophages can prevent *M. tuberculosis* from replicating. This enzyme is produced when macrophages are infected with *M. tuberculosis*. It has also been reported that PHOX induction causes ROS levels to rise. The generated mycobacterial catalase KatG, which contains catalase and peroxidase activity, inactivates ROS, making *M. tuberculosis* relatively resistant to being killed by them. Infection with *M. tuberculosis* also increases heme oxygenase (HO-1) expression in mouse macrophages and other host cells, most likely through the TNF-α signaling pathway. The two-component system DosS/T appears to be the mechanism by which CO, one of the reaction products of HO-1, and NO produced by iNOS promote transcription of the Mtb dormancy regulon. In addition, elevated amounts of host cell proteins necessary for the production of ROS, such as the neutrophil cytosolic factor 1 (NCF1 or p47) and the p67phox component of NADPH oxidase, are detected. The production of the antioxidant Mn-dependent superoxide dismutase, which quenches ROS and hydrogen peroxide, is also increased (Tounta et al., 2021).

The unique lipids found in mycobacterial cell walls are crucial to *M. tuberculosis* pathogenic processes. 166 different macrophage proteins were differently expressed when *M. tuberculosis* lipids were exposed to macrophage-like cells in the laboratory. Although the roles of these proteins are not fully defined, a significant fraction of the differentially expressed proteins (14%) appeared to be involved in metabolism (Tounta et al., 2021).

As human tuberculosis progresses, the caseous pulmonary granuloma, which contains a core of infected macrophages, develops. Prior studies have revealed an abundance of lipid species, including cholesterol, cholesterol ester, and triacylglycerol in these infected cells. Transcriptome analyses of such TB granulomas showed a considerable upregulation of genes involved in the sequestration, degradation, and synthesis of host lipids. The fact that several of the upregulated genes are likewise increased by TNF-α suggests that this response may be brought on by the chronic inflammation primarily generated by *M. tuberculosis* cell wall components. If *M. tuberculosis* colonizes the granuloma in a latent state, the accumulating lipids may provide the necessary carbon supply for the bacteria (Tounta et al., 2021).

Using^1^H-NMR-based metabolite profiling studies from the mouse and guinea pig models infected with *M. tuberculosis*, researchers were able to get detailed information on the metabolic changes of the host. The lung, liver, and spleen tissues that were evaluated in the infected animals showed qualitatively similar alterations of the key catabolic and anabolic chemicals. The lung, the primary site of *M. tuberculosis* infection, exhibits the most significant results quantitatively. It has been observed that lactate increases, whereas levels of glucose, glycogen, NAD, and NADP level decline. This indicates higher glucose consumption via the glyoxylate shunt (GL) and the pentose-phosphate pathway. This further supports the decreased levels of the TCA cycle intermediates such as oxaloacetate and fumarate, thereby decreased TCA cycle activity. However, the concentration of succinate, another intermediate of the TCA cycle showed increased levels (Tounta et al., 2021).

The increased glutaminolysis in mitochondria brought on by the oxidative stress experienced during *M. tuberculosis* infection may be the cause of this elevated level of succinate. As an alternative, *M. tuberculosis* may secrete succinate, which has been discussed above. This may induce increased lipolysis in the host cells. Furthermore, the amount of several amino acids also rises in the tissues that were examined and even in the serum of the *M. tuberculosis*-infected mice, indicating accelerated amino acid catabolism and/or proteolysis. The “anabolic block” seen in TB patients may be connected to this metabolic alteration (Tounta et al., 2021).

Additionally, elevated levels of a number of pyrimidine and purine nucleotide biosynthesis intermediates were found in the infected lung. The infected lung and spleen were also found to have higher levels of the antioxidant glutathione (GSH), another metabolic response to protect against induced oxidative stress. Similar patterns of metabolites are found in the lung tissues and serum of infected guinea pigs when metabolite profiling is performed (Tounta et al., 2021).

Overall, *M. tuberculosis* infection leads to substantial metabolic host responses that affect the host’s ability to fight off the infection as well as increased lipid metabolism, glucose uptake, and proteolysis. These metabolic host responses, which were seen *in vivo* mouse and guinea pigs, appear to support intracellular replication of *M. tuberculosis*. Most of the identified metabolic host responses appear to be significantly influenced by the distinct mycobacterial cell wall components (Tounta et al., 2021).

##### 4.1.2.2 Leprosy

*Mycobacterium leprae* is the etiological agent of leprosy. The global burden of leprosy was 28,800 DALYs in 2019 (Table 2). The sensory system may become scarred or impaired as a result of leprosy. Additionally, it leads to ulcers, profound atrophy, or profound sensory impairment (Santacroce et al., 2021).

##### 4.1.2.3 Urinary tract infection (UTI)

Approximately 150 million cases of urinary tract infections (UTIs) occur each year, making it one of the most prevalent bacterial infections worldwide (Mann et al., 2017). UTIs may develop as community-acquired or during health-care-related treatment. Urinary tract infection (UTIs) is one of the most prevalent bacterial infection and, predominantly affect women (Conover et al., 2016). A wide range of Gram-positive and Gram-negative bacteria such as *Escherichia coli*, *Proteus mirabilis*, *Klebsiella pneumoniae*, *Staphylococcus saprophyticus*, and *Enterococcus faecalis* have been linked to UTIs. However, up to 75% of all cases and 95% of cases that are community-acquired are due to Uropathogenic *E. coli* (UPEC), which is the main causative agent of the disease (Mann et al., 2017).

The gut microbiome is the source of UPEC, a pathotype of extraintestinal pathogenic *E. coli* (ExPEC). UPEC rarely creates any health issues in the colon. However, UPEC can spread and colonize in the urinary tract and the bloodstream. The organism secretes toxins, which can trigger an infection in the host. It is evident that UPEC’s ability to utilize nutritionally diverse habitats, including the intestines, urine, bladder, kidneys, and bloodstream, significantly contributes to the disease’s etiology. Because UPEC metabolism is tightly controlled and extremely responsive to the availability of nutrients, it can survive in a wide variety of environments that are both competitive and in fluctuating conditions. UTIs are typically initiated by UPEC that contaminate, colonize, and migrate into the urethra and bladder lumen. There is evidence that the majority of human UTIs are caused by UPEC strains that infiltrate the bladder epithelium and go through an intracellular infection cycle. The infection cycle is a complicated process that includes epithelial attachment, invasion of host cells, and intracellular proliferation. Ultimately, the bladder epithelial cell ruptures, causing infection to spread and infect neighboring epithelial cells. Lower urinary tract infections have the potential to spread to the kidneys and enter the bloodstream, leading to urosepsis, which can be fatal. As UPEC travels between the intestinal lumen and the urinary tract, they need to quickly adapt to new environments. One of the main sources of nutrients that UPEC receives in the urinary system is urine. It is yet unclear how the metabolic variety of urine lead to the development of UPEC in the urinary tract (Mann et al., 2017).

Urinary metabolites are more likely trigger the genetic and metabolic adaptation mechanisms of UPEC in order for the bacteria to survive and spread infection inside the urinary system. Aerobic respiration and carbon metabolism are critical for survival during intracellular UPEC colonization of the bladder and kidneys. Mice models infected with UPEC mutants showed that upregulation of the gluconeogenesis and the tricarboxylic acid (TCA) cycle is required for the colonization and survival of the pathogen during bladder infection. These observations showed that the organism can adapt to different nutrient availability within the host environments (Mann et al., 2017).

UPEC also possesses a variety of iron uptake and transport systems during the intracellular infection cycle. These are iron-chelating sideraphores and hemophores. The host can withhold free iron through the iron-binding proteins like lactoferrin, ferritin, transferrin, ovalbumin, and siderocalin. Siderocalin has high affinity to the *E. coli* siderophore enterobactin. Siderocalin has low binding affinity to the siderophores that are associated with UPEC strains. As a result, these provide evolutionary advantage for the UPEC to scavenge host iron (Alteri and Mobley, 2015; Mann et al., 2017). Other than animal model studies, LC-MS analysis of UTI patients’ urine sample revealed a variety of diamines, polyamines, and acylated conjugates (Table 3) These molecules have served as biomarkers for urine infection for over 3 decades (Satink et al., 1989; Lussu et al., 2017).

#### 4.1.3 Parasite infection and host metabolism

##### 4.1.3.1 Malaria

*Plasmodium vivax* and *Plasmodium falciparum* are the parasites that cause malaria. The disease is transmitted by mosquitoes. The disease, at its acute stage can lead to death and disability. Children who survive from cerebral malaria suffer from neurological effects and the effects are severe. In 2019, 643,000 people died from malaria. Of these fatalities, 356,000 occurred in children under 5 years old (Nagana Gowda and Raftery, 2013). There has been interest in using metabolic markers to support non-invasive disease diagnosis and the prognosis of disease severity. Significant changes in metabolic profiles based on molecules like amino acids and lipids have been detected when MS was used to diagnose P. falciparum infection from plasma samples. GC/MS was used to identify disease stages in pediatric plasma samples that were infected by the parasite. Increased amounts of 3-hydroxybutyric acid and fatty acids were detected in the samples. An increased level of valine was detected which had the potential connection between the severity of the illness and an increase breakdown of hemoglobin. A decreased level of alanine and pyruvate, which is connected to gluconeogenesis was observed (Tounta et al., 2021).

Other than plasma samples, in a separate study, high-performance liquid chromatography-high resolution mass spectrometry (HPLC/HRMS) was used to detect potential urine biomarkers in both healthy individuals and infected individuals. Following antimalarial therapy, the observed elevations in metabolite levels decreased. The differences in 1,3-diacetylpropane, N-acetylputrescine, and N-acetylspermidine levels between patients and controls make these molecules promising candidates to be used as biomarkers of infection. An abnormal level of amino acids and their metabolites such as threonine and trimethyl-L-lysine was detected too. These metabolic reprogramming are also related to kidney injury (Tounta et al., 2021).

### 4.2 Therapeutics

The infection triggers a complex host response. The establishment and severity of the disease are significantly influenced by the virulence of the infecting pathogen. Host genetic variations play a key role in the vulnerability to disease and the duration of an infection. Genome-wide association studies (GWAS) have made it possible to identify biomarkers linked to pathogens such as the human immunodeficiency virus (HIV), hepatitis C virus (HCV), dengue virus, malaria, and *Mycobacterium tuberculosis*. GWAS are a powerful tool for establishing a causal relationship between genetic polymorphisms and specific diseases (Yu et al., 2021). Pharmacogenomics is the study of how a person’s genetic make-up affects their response to medications and intends to guide the right choice and dosing of therapy. A large portion of the analyses in pharmacogenomics is focused on SNPs in order to comprehend the effects of single nucleotide polymorphisms (SNPs) on drug metabolism and disposition. SNPs that affect the expression and/or activity of metabolizing enzymes and drug transporters and alter therapeutic efficacy and safety profiles are of special interest. Pharmacogenomics uses a star nomenclature system (for example, CYP2B6*6) to designate common genetic variants that may have clinical importance. *1 is commonly associated with the wild-type allele in this naming system (Yu et al., 2021).

Pharmacogenomics is emphasized in therapies that are approved for the treatment of infectious diseases on an individual basis. Through treatment tailoring and individualization based on numerous intrinsic (e.g., organ dysfunction, genotype) and extrinsic (e.g., diet, drug interactions) aspects, clinical pharmacology plays a key role in precision medicine and drug discovery. Pharmacogenomics can be useful in detecting drug responders and non-responders, preventing side effects, and adjusting drug dosage. The primary objective of infectious disease management aims at the identification of genetic elements of the pathogen(s). The patient’s pharmacogenetic and/or immunogenetic profiles, however, may give the physician additional insights to help the patient effectively combat infection (Bissonnette and Bergeron, 2012). Drugs can be labelled with genomic biomarkers information and describe the events associated with drug exposure and clinical response, adverse events, genotype-specific dosing, mechanism of drug action, drug disposition and target genes with polymorphism (Bissonnette and Bergeron, 2012; Yu et al., 2021). Table 4 shows some of the drugs that are FDA approved and used for infectious disease treatment at the personalized level.

**TABLE 4:** Selected drugs approved for treating infectious diseases in a tailored approach.

Pathogen	Disease	Drug	Brand	Biomarker	Affected subgroups	Description of gene-drug interaction	Target protein/molecule	Pathways/others	Status	References
Virus	HIV	Abacavir	Ziagen®	HLA-B	*57:01 allele positive are hypersensitive to the medication.	Results in higher adverse reaction risk (hypersensitivity reactions). Patients positive for HLA-B*57:01 should not be treated with abacavir. (FDA recommends therapeutic management)	Reverse transcriptase	Nucleoside pathway	Marketed	Personalized Medicine Coalition (2020)
		Atazanavir	Reyataz®	UGT1A1	Patient’s with mutation in the gene suffer from hyperbilirubinemia, especially those with Glibert’s syndrome who carries mutation in the gene.		HIV protease	Protease inhibitor		
		Dolutegravir	Tivicay®	UGT1A1	Poor metabolizers. Decreased drug clearance was observed in patients with mutation in the gene. Neuropsychiatric adverse events were observed in some patients also.	Results in higher systemic concentrations. (There is evidence of a potential impact on pharmacokinetic properties of the drug. There is no demonstrated relationship between the safety or reaction to the related medicine and genetic variants or genetic variants inferred phenotypes).	Integrase	HIV integrase inhibitor		
		Efavirenz	Sustiva® and Stocrin®	CYP2B6	Poor metabolizers. Mutation in the gene affects drug metabolism and clearance from the body.	Increases systemic concentrations and the likelihood of adverse reactions (Pharmacogenetic relationship indicates a potential effect on response or safety)	Reverse Transcriptase	Reverse Transcriptase inhibitor		(Personalized Medicine Coalition, 2020; Yu et al., 2021)
		Maraviroc	Selzentry®	CCR5, CYP3A5	*3/*3 and *1/*1 loss of function allele, poor metabolizer.	In homozygous dysfunctional populations, higher plasma levels and reduced clearance is observed.	CCR	CCR antagonist. Blocks viral gp120 from interacting with co-receptor.		Personalized Medicine Coalition (2020)
		Raltegravir	Isentress®	UGT1A1	*28/*28 (poor metabolizers)	Increases systemic concentrations. (There is evidence of a possible effect on pharmacokinetic properties. There is no demonstrated relationship between the safety or reaction to the related drugs and genetic variants or genetic variants inferred phenotypes.).	Integrase	Integrase inhibitor		
	Hepatitis C	Boceprevir	Victrelis®	IFNL3 (IL28B)		One of the key predictors of HCV clearance is IL-28B polymorphisms.	HCV Protease	Protease inhibitor		(Matsuura et al., 2014; Personalized Medicine Coalition, 2020)
		Daclatasvir	Daklinza®							
		Dasabuvir, ombitasvir, paritaprevir, and ritonavir	−							
		Ombitasvir, paritaprevir, ritonavir	−							
		Peginterferon alfa-2a	Pegasys®							
		Peginterferon alfa-2b								
		Elbasvir and grazoprevir	Zepatier™							
		Ribavirin	−				−	Anti-viral		
		Simeprevir	−				HCV protease	Protease inhibitor		
		Sofosbuvir	Sovaldi®				DNA/RNA Synthesis	DNA Damage		
		Sofosbuvir and velpatasvir	Epclusa®				−	Protease inhibitor		
		Sofosbuvir, velpatasvir, and, voxilaprevir	Vosevi®				-	-		
		Telaprevir	Incivek®				−	-		
		Ledipasvir and sofosbuvir	Harvoni®				−	−		
	Leprosy	Dapsone	−	G6PD	Patients with Glucose 6-Phosphate Dehydrogenase (G6PD) deficiency suffer from severe haemolysis if treated with Dapsone.		−	Antibiotic		(Aftab et al., 2016; Personalized Medicine Coalition, 2020)
	Urinary tract infections	Nalidixic acid	−	G6PD			Topoisomerase	DNA Damage		Personalized Medicine Coalition (2020)
		Sulfamethoxazole and trimethoprim	Bactrim ®	G6PD; NAT	NAT is related to poor metabolizers	May result in higher adverse reaction risk (Pharmacogenetic association data indicate a potential impact on safety or response)	Nucleic acid synthesis pathway.	Antibiotic		
		Nitrofurantoin	Furadantin®	G6PD			−	Antibiotic		
	Tuberculosis	Isoniazid, pyrazinamide and rifampin	Nydrazid®, Rifater®, Rifadin®	NAT	NAT is related to poor metabolizers	May result in higher systemic concentrations and adverse reaction risk. (Pharmacogenetic association data indicate a potential impact on safety or response)	-	Anti-infection		
Parasite	Malaria	Primaquine	−	G6PD; CYB5R		People with G6PD require higher dose of the drug.	−	−		(Personalized Medicine Coalition, 2020; Visvikis-Siest et al., 2020)
		Quinine sulfate	Qualaquin®	CYP2D6; G6PD			−	−		
		Tafenoquine	−	G6PD			−	−		
		Chloroquine	−	G6PD			Autophagy, ATM/ATR	PI3K/Akt/mTOR		
		Hydroxychloroquine	Plaquenil®	G6PD			Autophagy	Autophagy		

Note: HLA-B = Human leukocyte antigen-B; UGT1A1 = UDP, Glucuronosyltransferase Family 1 Member A1; CYP2B6 = Cytochrome P450 Family 2 Subfamily B Member 6; G6PD = Glucose-6-phosphate dehydrogenase, IFNL3 = Interferon Lambda 3; NAT = N-acetyltransferase; CYB5R = Cytochrome b5-related protein; CCR5 = C-C chemokine receptor type 5; CYP2D6 = Cytochrome P450 2D6; CYP2C19 = Cytochrome P450 2C19; P450 = Cytochromes P450; CYP17 = Cytochrome P450 17A

### 4.3 Diagnostics

#### 4.3.1 Point-of care tests (POCTs)

POCTs belong to *in vitro* diagnostic tests that use biological samples such as blood, sweat, urine or tissue in order to diagnose a patient (Mukherji and Mondal, 2017; Castelli et al., 2022). The tests are performed in a controlled environment outside a living organism. Each year, thousands of new POCT devices are launched and medical professionals are increasingly employing these for early disease diagnosis. POCT offers advantages as it is a fast, sensitive, and inexpensive diagnostic that enables more accurate patient stratification, diagnosis, and treatment options (Mukherji and Mondal, 2017; Castelli et al., 2022).

POCT combines expertise in advanced manufacturing and analytical chemistry (Mukherji and Mondal, 2017; Castelli et al., 2022). Some POCTs can detect metabolites from a specific metabolic pathway. POC has evolved in terms of sensitivity, accuracy, and adaptability for use in the individualized diagnosis of infectious diseases due to miniaturization, smartphone-based sensing assays, and lab-on-a-chip (LOC) (Mukherji and Mondal, 2017; Castelli et al., 2022). The POCT testing can be multiplexed or segmented into dipsticks, lateral flow immunoassays (LFIA), and microfluidics (Mukherji and Mondal, 2017; Castelli et al., 2022). The basis for LFIA involves the interaction of a sample with labelled antibody that has been pre-loaded on a strip of polymer, nitrocellulose, papers or other materials (Mukherji and Mondal, 2017; Castelli et al., 2022). Conversely, microfluidic diagnostic allows for precise regulation over the rate of flow of samples and reagents through the microchannel, leading to the separation and detection of the intended analyte. Each POC testing system has a sample handling platform and a signal transduction unit that is unique to the system. Proteins, disease-specific biomarkers, and cell density are the target analytes, and the testing platforms are integrated with various sensing mechanisms including electrochemical, colorimetric, fluorescence, and spectroscopy (Mukherji and Mondal, 2017; Castelli et al., 2022). For POCT, it is critical to choose appropriate specific biomarkers (Mukherji and Mondal, 2017; Castelli et al., 2022). Table 5 shows some of the POCT that are approved for the detection of different pathogens and commercially available.

**TABLE 5:** List of selected POCT for Infectious diseases that are commercially available.

Disease	POC device name	Test approach	Biomarker	Sample type (metabolic pathway)	Result time (minute)	Storage temperature (◦C)	Benefit	References
Dengue	SD Bioline Dengue Duo (Dengue NS1 Ag + IgG/IgM)	Lateral flow test strips (Antibody based, qualitative test)	Dengue NS1 Ag + IgM/IgG	Serum/plasma/whole blood (Energy metabolism-ATP production)	15–20	2–30		(Mukherji and Mondal, 2017; Castelli et al., 2022)
	Panbio Dengue Early Rapid Kit	Lateral flow (cassette) (Antibody based, qualitative test)	Dengue NS1 Ag	Human serum, plasma or whole blood	15–20	2–30		
	Dengue NS1 Rapid test (Strip)	Membrane-based immunoassay (Antibody based, qualitative test)	Dengue NS1 Ag	Human serum	30	30		
	CareStart™ Dengue Combo (NS1+IgM/IgG)	Lateral flow (Antibody based qualitative test)	Dengue NS1 Ag + IgM/IgG	Serum, plasma or whole blood	15–20	1–30		
	Bhat Bio-SCcan® Dengue NS1 & IgG+IgM Combi Card Test	Immunochromatographic assay	Dengue NS1 Ag + IgM/IgG	Human serum and plasma	20	2–30		
	Standard Q Dengue Duo test	Immunochromatographic assay	Dengue NS1 Ag + IgM/IgG	Human serum, plasma, and whole blood	15–20	1–40		
Tuberculosis	Alere Determine™ TB LAM Ag	Lateral flow	Lipoarabinomannan	Urine (glycolysis, amino acid)	25	2–30	Screening tool for early detection of TB is useful in numerous low-income or resource-poor nations or settings where tuberculosis is not under control.	
	EasyNat TB-CPA Diagnostic Kit	Isothermal amplification- lateral flow assay	*M. tuberculosis* DNA	Sputum (Pyruvate metabolism)	120	−		
	Tuberculosis IgM/IgG Rapid Test	Sandwich lateral flow chromatographic immunoassay	TB IgM/IgG	Human serum or plasma (glycolysis, amino acid metabolism)	10	2–30		
Hepatitis B virus	VIKIA® HBs Ag	Immunochromatographic or lateral flow	HBs Ag	Human serum, plasma, or whole blood	15	4–30	HBV causes chronic viral hepatitis. Patients can be co-infected with HBC and HAV. Early and rapid detection can help clinicians start treating patients immediately.	
	HBsAg Rapid Test (strip)	Lateral flow chromatographic immunoassay	HBsAg	Human serum or plasma	15	−		
	Alere Determine™ HBsAg	Lateral flow	HBsAg	Human serum, plasma, or whole blood	15	2–30		
	EuDx TM-HE (A, B, C) KIT	Immunochromatographic method	HAV IgM, HBsAg, and anti-HCV	Serum sample	15	−		
	SD Bioline Anti-HBsAg	Immunochromatographic method	HBsAb	Human serum or plasma	−	2–30		
HIV/AIDS (Restricted to clinical laboratories)	Alere Determine™ HIV- 1/2	Lateral flow	−	Serum, plasma, or whole blood	15	2–30	Rapid HIV/AIDS patient screening improves point of care and patient management.	
	Chembio Dual Path Platform (DPP®) HIV 1/2	Immunochromatographic test	HIV-1/2 Ab	Oral fluid	10	2–30		
	OraQuick Advance®	Immunochromatographic test	HIV-1/2 Ab	Oral fluid	20	−		
	HIV 1/2 AntibodyTest Strip	Lateral flow immunoassay	HIV-1/2 Ab	Whole blood, serum, or plasma	10	2–30		
	INST® HIV-1/HIV-2 Antibody Test	Immunofiltration “flow-through” approach	HIV-1/2 Ab	Whole blood, fingerstick blood, serum or plasma	1	−		
Others								
	Acro Biotech COVID-19 15 min RAPID POC test	Lateral flow chromatographic immunoassay	SARS-CoV-2 IgG and IgM	Whole blood, serum, or plasma	15	2–30		

Note: NS1 = Non-structural protein of dengue.

#### 4.3.2 Companion diagnostics

A companion diagnostics (CDx) is a medical device that is used in conjunction with therapeutic drugs to assess the effect and suitability of drugs on the human body (Waldman and Terzic, 2015). The CDx tests are biomarker tests that help in drug usage decision-making. The FDA classifies these tests as class III *in vitro* diagnostic tests (Jørgensen, 2021). Companion diagnostics and drugs are used to exclude or select patient groups according to their response to therapy. These devices enable healthcare professionals to make decisions and analyzing improvements in the treatment. CDx is thus also known as a targeted and predictive assay device (Jorgensen, 2015). The first-generation CDx assays detect a single biomarker. The detection techniques are PCR, FISH, and ChIP-seq. However, the FDA has approved multiplex CDx assays that use the next-generation sequencing (NGS) platform. These devices can also detect drug or therapy resistance in patients. The rising incidence of cancer and the importance of personalized medicine are driving the global market size of companion diagnostics to increase. Infectious diseases are occasionally regarded as ideal examples of personalized medicine applications. The usefulness of biomarkers connected to the immune response, infectious disease susceptibility, host-microbiota interactions, or responsiveness to antimicrobial medication treatment, however, is gradually changing this perception (Bissonnette and Bergeron, 2012). For the molecular management of infections, personalized medicine for infectious diseases has clear advantages. In fact, the use of a personalized medicine approach could be conceptualized as a bimodal process aiming to decipher the clinically-relevant genomic components of the patient and the disease-associated pathogen(s) in order to choose and optimize the course of treatment for acute life-threatening diseases. Tables 6, 7 show some of the FDA-approved companion diagnostics for infectious diseases. Mutational genes of the pathogen can be detected using the CDXs assay, and clinicians can make the appropriate treatment decisions (Nordström and Lewensohn, 2010).

**TABLE 6:** List of selected companion diagnostics (CDx) for detection of drug resistant viruses.

Disease/Pathogen	Number of known mutations	Targeted drugs	Test type	Test name	Detection technology	Sample type	Status	References
HIV resistance testing	57	20 (-NNRTI, -NRTI, -INI, -PI)	Genotype	ViroSeq, (Abbott Molecular, Chicago, IL, United States)	RT-PCR, Sanger sequencing	Plasma	Kit. FDA approved.	(Dailey et al., 2020; US Food and Drug Administration, 2020)
				Sentosa SQ, (Vela Diagnostics, Singapore)	RT-PCR, NGS	Plasma	Kit. FDA Approved.	
HCV resistance testing	6	7 (Mavyret, Vosevi, Epclusa, Zepatier, Daklinza, Harvoni, Sovaldi	Genotype	Sentosa SQ HCV Genotyping Assay (Vela Diagnostics)	RT-PCR, NGS	Serum or plasma	Kit. FDA approved	
				GenoSure HCV (Monogram Biosciences)	RT-PCR, NGS	Serum or plasma	LDT	
HBV resistance testing	9	6 (Tenofovir disoproxil, Tenofovir alafenamide, Entecavir, Telbivudine, Adefovir dipivoxil, Lamivudine)	Genotype	−	PCR, Sanger sequencing	Plasma or serum	LDT. Available through commercial laboratories	
				−	PCR, multicolor melting curve analysis	−	LDT	
				−	PCR, Ultra Deep Pyrosequencing	−		
				−	PCR, Microarray	−		
Influenza	4	4 (Ganciclovir, Valganciclovir, Foscarnet, Cidofovir)	Genotype	Roche 454 Life Sciences (Branford, CT, United States)	NGS/pyrosequencing	Nasal and nasopharyngeal	LDT	
				−	RT-PCR	−	LDT	

Note: NNRTI: Non-nucleoside reverse transcriptase inhibitor; NRTI: nucleoside reverse transcriptase inhibitor; INI: integrase inhibitor; PI: protease inhibitor; HIV, human immunodeficiency virus; HCV, Hepatitis C virus; HBV, Hepatitis B virus; HPV, human papilloma virus; CMV, human cytomegalovirus; HSV-1, Herpes simplex virus subtype 1; HSV2, Herpes simplex virus subtype 2.

**TABLE 7:** List of selected companion diagnostics (CDx) to detect drug resistant genes of bacteria.

Pathogen	Test name/manufacturer	Sample type	Detection technology	Mutational genes/Targets	References
Bacteria					(Dailey et al., 2020; US Food and Drug Administration, 2020)
*Mycobacterium tuberculosis*	COBAS® MTB-RIF/INH (Roche Molecular Systems, Pleasanton, CA, United States)	Sputum or bronchial alveolar lavage	Nucleic Acid Amplification	Mutations in the *rpo*B gene associated with Rifampicin-resistance; mutations in the *kat*G and *inh*A genes linked to isoniazid-resistance.	
	Xpert® MTB/RIF Ultra (Cepheid, Sunnyvale, CA, United States)	Sputum or bronchial alveolar lavage		MTB and rifampin resistance mutations	
	RealTime MTB RIF/INH (Abbott, Chicago, IL, United States)	Sputum or bronchial alveolar lavage		Rifampicin (RIF) and isoniazid (INH) resistance targeting up-stream promoters of *rpo*B, *kat*G and *inh*A genes.	
	GenoType MTBDRplus VER 2.0 (Hain LifeScience, Gmbh, Nehren, Germany)	Pulmonary specimens and/or liquid or solid culture samples		*rpo*B gene that confers rifampicin resistance; *kat*G gene resistant to high level isoniazid; the promoter region of the *inh*A resistant to low level isoniazid.	
	GenoType MTBDRsl VER 2.0 (Hain LifeScience, Gmbh)	Pulmonary specimens and/or liquid or solid culture samples		Genes resistance to fluoroquinolones, aminoglycosides/cyclic peptides and ethambutol	

### 4.4 Drugs targeting host metabolic pathways for the treatment of infectious disease

While metabolomics can be used as biomarkers of disease diagnosis, drugs targeting the host metabolic pathway also possess potential benefit to manage infectious disease. Antimicrobial resistance has grown widely, necessitating the development of additional therapies in addition to new antibiotics. The development of a wide spectrum of host-directed medicines that target and modify biological pathways to achieve a successful therapeutic treatment outcome has been fueled by a renaissance in scientific research methodologies targeting host variables over the past 2 decades rather than directly targeting pathogen components. Host-directed therapies that have wide-ranging effectiveness may also be helpful in treating infectious diseases that have the potential to become epidemic and are related to high mortality cases. The unique advantage of host-directed treatments is that they can stop or slow the emergence of antibiotic resistance (Zumla et al., 2020). Table 8 shows examples host-directed therapies that are used to target the metabolic pathway for a number of infectious diseases caused by viruses, bacteria and parasite.

**TABLE 8:** List of selected antimicrobial drugs targeting host metabolic pathways.

Pathogen type	Disease/Species name	Drug name	Host factor targeted	References
Virus	Hepatitis C virus (HCV)	Mycophenolic acid and Ribavirin	Inosine monophosphate dehydrogenase (IMPDH)	Mahajan et al. (2020)
	DENV	DFMO and Diethylnorspermine	Host Polyamine synthesis pathway	
		Celgosivir	Alpha-glucosidase I inhibitor (hostdirected glycosylation)	
	SARSCoV-2	Sanglifehrin A	IMPDH	
		Ribavirin	IMPDH	
		Mycophenolic acid	IMPDH	
		Merimepodib	IMPDH	
		Loratadine	Sodium-dependent neutral amino acid AT2 from SLC6A15 gene	
	Dengue	Cerulenin, C7594, pravastatin, U18666A, Medica, TOFA, GGTI (geranyl geranylationinhibitor), Lovastatin, 25-Hydroxycholesterol, Fluvastatin with Peg-IFN/Ribavirin, AM580, PF-429242	Fatty acid synthesis inhibitor	
	SARS-CoV-2	cPLA2α, PCSK9, A939572, Fingolimod, C75, Cerulenin, Fibrates, Triacsin C	Inhibits lipid biosynthesis	
	HCV	Quercetin	GLUT1 (glucose transporter 1)-Glycolytic pathway	
		Silibinin	GLUT4 (glucose transporter 4)-Glycolytic pathway	
		LY294002	PI3K (phosphatidylinositol 3-kinases)-Glycolytic pathway	
	Dengue	Quercetin	GLUT1-Glycolytic pathway	
		Silibinin	GLUT4-Glycolytic pathway	
		Luteolin	HEK2 (hexokinase 2) -Glycolytic pathway	
	SARS-CoV-2	Fasentin, Phloretin	GLUT2-Glycolytic pathway	
		Ritonavir	GLUT4-Glycolytic pathway	
		Silybin/Silibinin, STF-31	GLUT1-Glycolytic pathway	
		Phloridzin	SGLT1-Glycolytic pathway	
		Dapagliflozin	SGLT2-Glycolytic pathway	
		Metformine, Resveratrol, Ivermectin	AMPK activator-Glycolytic pathway	
	H1N1 influenza A virus	2DG and BrPa, 2DG and Oxamate	Glycolysis	Sumbria et al. (2021)
	HCV	Difluoromethylornithine (DFMO)	ODC1 (Ornithine Decarboxylase 1)-Polyamine pathway	Mahajan et al. (2020)
		Diethylnor spermidine (DENspm)	Polyamine metabolism	
		Ciclopirox (CPX), Deferiprone (DEF), and, GC7	Hypusination-Polyamine pathway	
	SARS-CoV-2	Difluoromethylornithine (DFMO)	ODC1 (Ornithine Decarboxylase 1)-Polyamine pathway	
	DENV	RBM10	SAT1-Polyamine pathway	
	SARS-CoV-2	Peptide-N-Glycosidase F (PNGase-F)	N-glycans-Polyamine pathway	
		Iminosugars Miglustat, Celgosivir and NNDNJ	ER a-glucoside I-Polyamine pathway	
		Deoxymannojirimycin, mannostatin A	α-mannosidase-Polyamine pathway	
		N-butyl deoxynojirimycin, N-nonyl deoxynojirimycin, castanospermine, celgosivir	α-glucosidase-Polyamine pathway	
	DENV	Castanospermine (CST) and deoxynojirimycin (DNJ)	α- Glycosidase-Polyamine pathway	
Bacteria	*Mycobacterium tuberculosis*	AM 92016, Benoxinate hydrochloride, BENZAMIL, DICHLOROBENZAMIL, FLECAINIDE, FLUNARIZINE, KB-R7943, LIDOCAINE, NICARDIPINE, NIGULDIPINE, Proadifen hydrochloride, QUINIDINE, TETRANDRINE, U-54494A, VERAPAMIL	Ion channel	Kuang et al. (2022)
		AG 957, AG1478, ALK inhibitor, allosteric AKT1/2 inhibitor, BML-265, Bohemine, EGFR inhibitor, GNF2, Abl inhibitor, GSK-3 Inhibitor II, PDGR inhibitor, PI3K/mTOR inhibitor, Diacylglycerol Kinase Inhibitor I, Diacylglycerol Kinase Inhibitor II, FTT, GF-109203X	Kinase inhibitor	
		Aspirin	Arachidonic acid metabolism in host. The drug is involved in dampening of TNFa-induced hyperinflammation to aid tissue repair and control burden of *M. tuberculosis.*	
		Zileuton	Arachidonic acid metabolism in host. Promotes reduced lung *M tuberculosis* burden and pathology	Kolloli and Subbian (2017)
		Vitamin D3	Induces autophagy of infected cells. VitD3 supplementation could augment faster recovery.	
		Phenylbutyrate	Promotes colocalization of LL-37 and LC3-1I in autophagosomes and restricts *M. tuberculosis* growth inside the macrophage	
Parasite	*Plasmodium falciparum*	Blockers of lipid transport (Bl Ts)	Targets scavenger receptor (SR)-81. Inhibits SR-81-mediated selective uptake of lipids from high-density lipoproteins	
		Nutlin-3	Targets MDM2. Prevents degradation of p53, promotes lipid peroxidation in infected hepatocytes	
		S8505124	TGF- receptor 1 is targeted. The drug inhibits the enzymatic activity of kinases involved in multiple cellular processes	
		Auphen	Targets aquaporin 3 (AQP3). The drug selectively and irreversibly inhibits glycerol transport by AQP3; effective against both liver and blood stages and against multiple human malarias.	
		lmatinib	Targets receptor tyrosine kinases. The drug inhibits erythrocyte band 3 phosphorylation, preventing parasite egress.	
		Erastin	Targets SLC7A11. The drug blocks host SLC7a11- GPX4 pathway to induce lipid peroxidation in *Plasmodium* infected hepatocytes.	

## 5 Current challenges

### 5.1 Technological challenges

Identification of robust markers is key to the success of personalized medicine. Metabolomics is used to identify biomarkers associated with disease diagnosis, prognosis and treatment. However, the complex chemical properties of different metabolites and overlapping roles in different metabolomic pathways requires the use of cross-validation platforms (Kennedy et al., 2018; Di Minno et al., 2022). Moreover, metabolites are modulated by genetic factors, signaling molecules, and proteins. Therefore, clinical interpretation of biomarkers requires the integration of data from various omics-based technological platforms. There are currently very few studies that combine all of the omics platforms (Kaddurah-Daouk et al., 2008). The host-immune response and infection-related co-morbidity induced by the pandemic strain, SARS-Cov-2, have been analyzed using integrated omics platforms. More research is needed to determine the root causes of vaccination relapse and antibiotic resistance in both the pathogen and the host’s immune system for other pathogens. Future research will need to combine both genomic and metabolomic data to find reliable biomarkers linked to a certain disease and its therapy (Kaddurah-Daouk et al., 2008).

### 5.2 Economic challenges on instrumentation

Instruments like NMR and MS are highly expensive. Instrument operation and data analysis demand for qualified personnel. For tailored diagnosis, there are numerous *in vitro* diagnostic kits that use data from metabolomics, genetics, and even proteomics. Companion diagnostics, which are *in vitro* diagnostics kits, are helpful since they can contribute to the detection of pathogenic organisms for which there is no vaccine available. Drawbacks include these tests are targeted assays that may miss other related organisms and that detection equipment may not be available or affordable in all parts of the world. Several companion diagnostics tests have been developed in laboratories (LDTs). Because of this, these are only economical in a specific region. On the other hand, POCTs, which are also *in vitro* diagnostics kits, are affordable in countries and regions with limited resources. However, for these, accurate biomarkers must be identified; otherwise, the patient will be at risk of receiving an incorrect diagnosis, which would necessitate expenditure at the personal level (American Association for Clinical Chemestry, 2021; Tolstikov et al., 2017; zhong, 2019).

In addition to NMR and MS, biobanks which are believed to be instruments for personalized medicine, are mostly accessible in high-income nations. Many nations throughout the world use context-based and automated biobanks. Human genome sequencing is being carried out in a number of nations as genetic information reduces the knowledge gap on a population’s genetic causes of illness. In an effort to better understand health and disease, the US has started analyzing the genomes of at least one million Americans who voluntarily provide their genetic data for research. Genomic profiling is being carried out in other nations as well. The 100,000 Genome Project in the United Kingdom, the One Million Genome Project in China, the Personal Genome Project in Canada, the FinnGen genomic sequencing and biobank project in Finland, the Qatar Biobank, and the Biobank Japan are a few examples. These initiatives are anticipated to offer the possibility of using huge amounts of information to comprehend human diversity on a global scale (MacDonald, 2020; Doxzen et al., 2022). Only a few underdeveloped nations can afford biobanking because of the high costs of the equipment, sample storage, and maintenance. For both rich and developing nations, dried blood spots (DBS) for newborn screening are a successful example of personalized medicine (Rudan et al., 2011).

### 5.3 Economic challenges in the health sector

**FIGURE 2:**
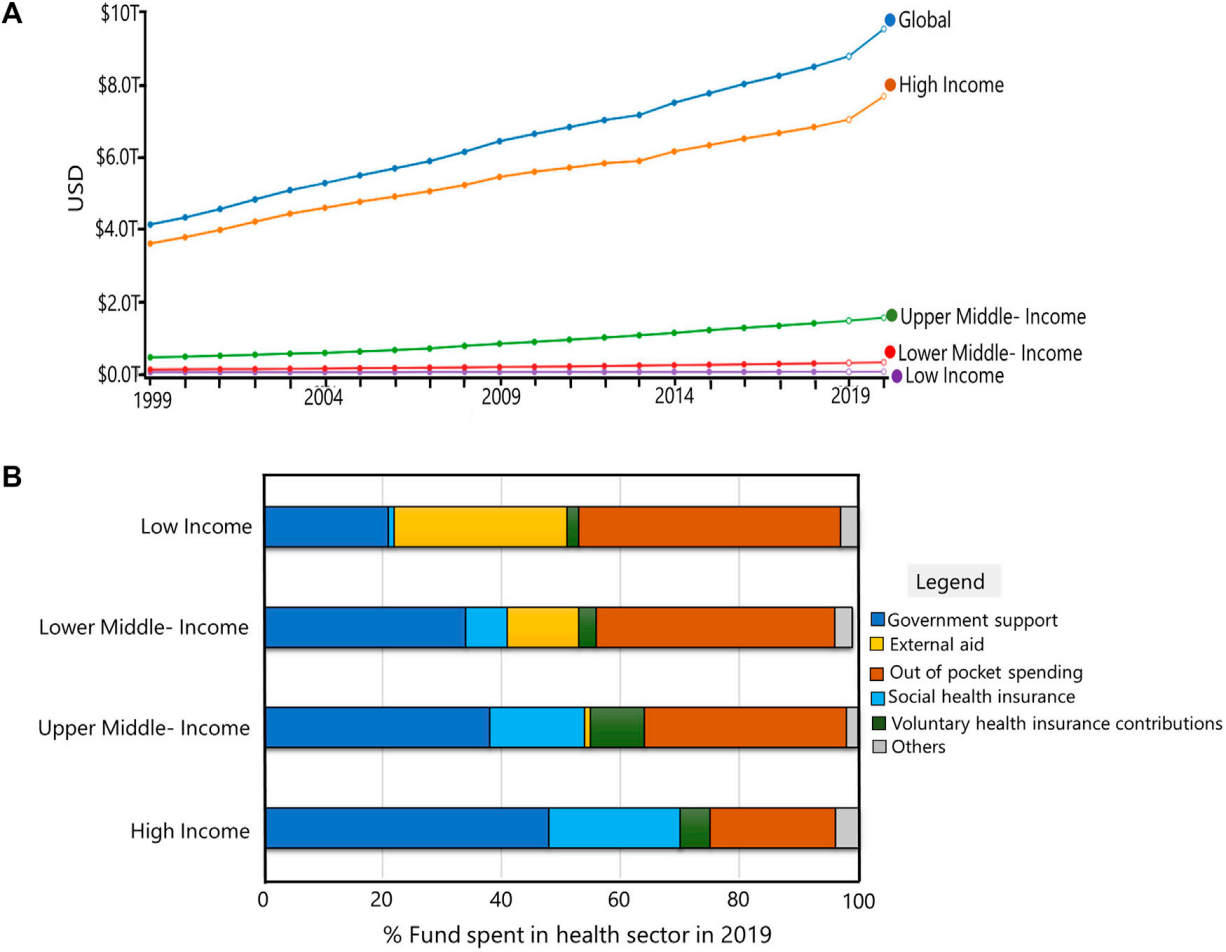
Global Financing in health sector: **(A)** Total spending in health sector for last 2 decades (USD). Global and countries categorized by World Bank based on GDP’s are shown. **(B)** Funds spent in health sector in high-income, upper-middle income, lower-middle income and low-income countries in 2019. Images of **(A)** and **(B)** were generated using the software provided at https://vizhub.healthdata.org/fgh/.

Prior to decisions regarding new drug formulation and treatment approaches, patient omics data must be made available at the clinical level. High-throughput technologies, which are quite expensive, are needed to accomplish this. This demands adequate funding in the healthcare field. Over the last 2 decades, the amount spent globally on healthcare has doubled. The health sector’s spending was US $ 8.9 trillion in 2019. There was also an increase in GDP, from 8.5% of GDP in 2000 to 9.8% of GDP in 2019. However, expenditure varies widely among nations (Figure 2). The World Bank has divided the world’s nations into four income segments based on total GDP: high-income, upper middle-income, lower middle-income, and low-income (WHO Global Health Expenditure Database, 2021). Government assistance for the health sector is higher in high income nations. On the other hand, high levels of personal expenditure are found in low-income nations. Low-income nations still can access outside aid, though. These, however, are insufficient because the development of disease- and patient-specific biomarkers and medications necessitates years of research, patent applications, regulatory agency approval, clinical trials, post-marketing evaluations, and investigations into drug responses that may not work (Alyass et al., 2015; Hartl et al., 2021; World Health Organisation, 2021).

### 5.4 Economic challenge at the industry level

The process of discovering new drugs based on the identification of biomarkers is expensive and time-consuming. Preclinical research, clinical testing, and post-market approval are all processes in the introduction of new medications. Drug efficacy and safety are assessed on volunteer patients during clinical trials. Moreover, only a small fraction of all medicines are tested and validated in clinical trials. Diagnostic assays or kits face the same difficulties as other medicines. It requires time to complete regulatory approval, clinical trials, and post-market validation. A product might be on the market for ten to 15 years of times. Personalized treatments or targeted tests are intended for small groups, hence it can be difficult for enterprises to balance cost and affordability (Alyass et al., 2015; Mathur and Sutton, 2017; American Association for Clinical Chemestry, 2021).

### 5.5 Challenges in the drug and diagnostics industry

Drug and diagnostics companies have different development timelines including phases of product development, returns on investment, customers, and regulatory requirements. Drugs are valued as products with high average values and are reimbursed accordingly. Diagnostics are considered services and compensated accordingly, usually at a substantially lower price. There are not many models, if any, available for valuing drug-diagnostic combinations. Patents provide protection for drugs, but there is less emphasis on intellectual property in the companion diagnostics sector because biomarkers are seen as being present in the cell. Since biomarkers are not inventions but rather preexisting components of cells, there is even argument as to whether they should be patentable at all (OECD Dir Sci Technol Ind, 2011).

### 5.6 Challenges to implementation of biomarkers in clinics

Multiple requirements must be fulfilled for a biomarker to be used in the clinic. The population must accurately represent the people where the test will be utilized. The study size should be selected carefully so that there is enough evidence to detect true biomarkers. However, this is challenging in metabolomics studies in the discovery phase because it is difficult to estimate the clinically significant differences required. The participants must be carefully controlled to avoid confounding variables. Patient-based outcomes rather than reduction of a disease biomarker must be chosen, and endpoints must be correctly judged in accordance with clinical trial guidelines. The biomarker needs to be accurately identified and measured precisely. For multi-center research in particular, collection, storage, preparation, and analysis processes must be completely harmonized to prevent artifactual results. The assays themselves need to go through rigorous Quality Control processes, particularly if batch to batch variation is anticipated a problem. The Metabolomics Quality Assurance and Control Consortium’s guidelines serve as an example of this. To make sure that results are reliable and generalizable, any novel biomarkers discovered in one study should be validated in a different cohort. When these criteria are met, this has the further advantage of supporting data for comparison and better biological interpretation. In addition, the biomarkers also need to be compared with gold standard methods and routine biochemistry tests to compare the turn-around time, availability, robustness, accuracy and precision. The turn-around time, availability, robustness, accuracy, and sensitivity of the biomarkers should also be compared to those of conventional procedures and standard biochemical assays. Finally, a health economics study is essential to determine whether any cost increases brought on by new tests are justifiable in terms of outcomes for patients (OECD Dir Sci Technol Ind, 2011).

## 6 Conclusion

Personalized medicine is a therapeutic option. Over the last decade, there has been an increase in reports of drug resistance and vaccine relapse. As a result, various drug regulatory bodies have coined the term “Personalized medicine” to describe the need for drug development based on a person’s genetic profile. However, this is still in its infancy and requires the involvement of multidisciplinary researchers to identify appropriate biomarkers related to disease cause and diagnosis. Profiling a patient’s genetic and metabolic fingerprints, as well as clinical interpretation, necessitates the use of omics technologies. Since metabolomics is one of the omics technology and provides information on genotype to phenotype changes, technologies that can ensure measurement and detection of all relevant molecules using untargeted and targeted approaches will play a crucial role in the identification of biomarkers associated with diseases and treatment response. In clinics, single metabolite measurement is performed using analytical and biochemical assays. However, use of NMR and MS enables metabolite detection from large number of samples. MS and NMR are the two major technological platforms used in clinical metabolomics.

Due to the complexity of the technology platforms, metabolomics studies that aims to identify biomarkers of disease necessitate the design of a workflow for accurate interpretation of the patient-related data. Selecting a single analytical platform does not offer information on all the chemicals present in the biosample as metabolites demonstrate a high degree of chemical diversity. Another hurdle in biomarker identification is handling and storing samples. These differ between countries and geographical areas. Implementation requires standard operating procedures (SOPs) for sample collection, storage, preparation, and analysis in clinics to maintain the consistency of the data. In addition to this, the platforms encounter significant difficulties as a result of ongoing improvements to the data pre-processing software. Software development for metabolome study requires the involvement of personnel from bioinformatics, statistics, and computational biology. The majority of software programs are used to preprocess, analyze, visualize, and manage data as well as databases. Despite these, numerous metabolites in the bio-sample remain unidentified.

**FIGURE 3:**
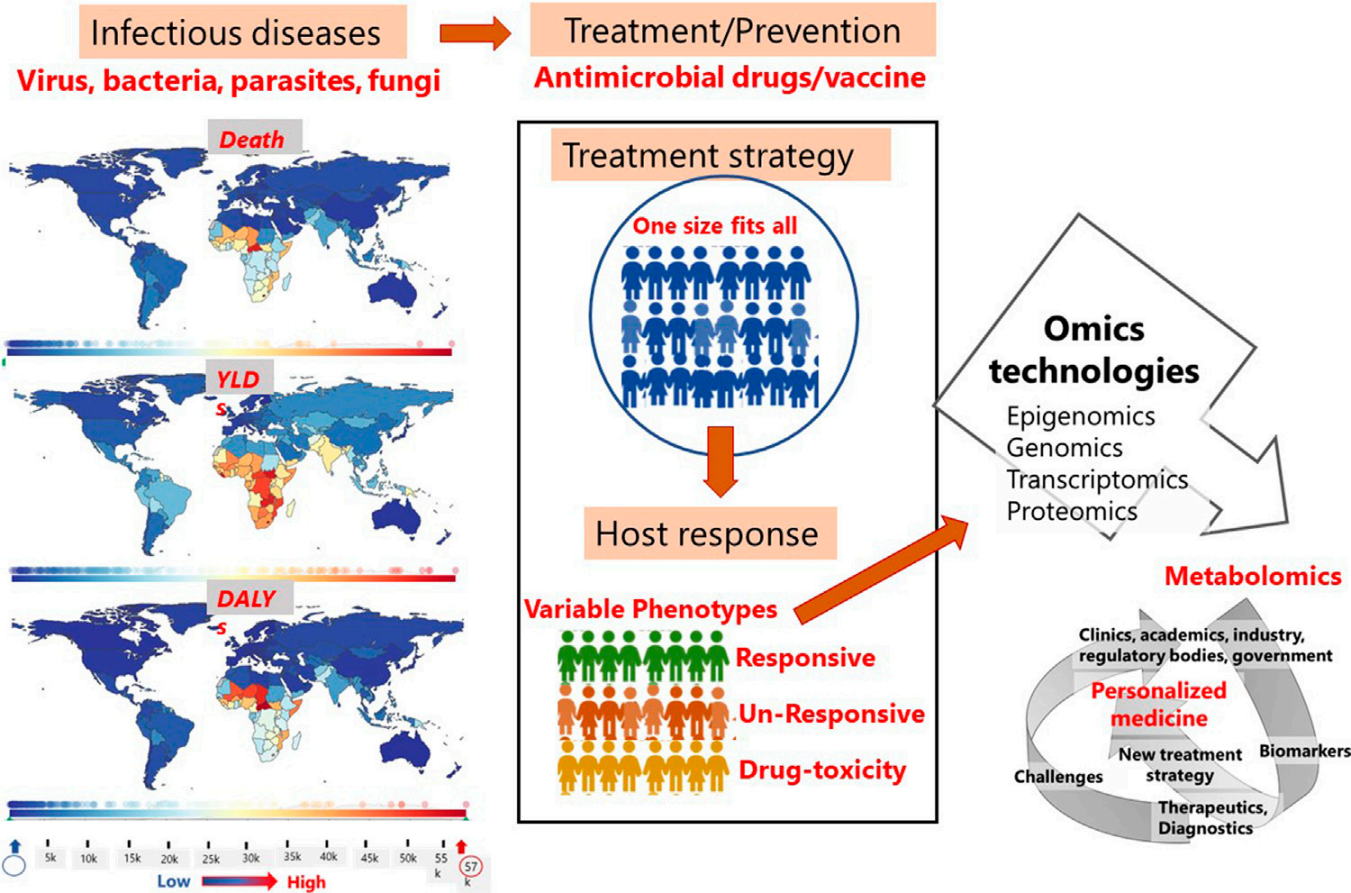
Factors and scope of metabolomics in personalized treatment of infectious diseases. Images on the statistics of death, YLDs and DALYs in different regions of the world due to infectious diseases was generated using the software provided at https://www.healthdata.org/results/gbd_summaries/2019/ World Health Organization (WHO). Note: YLDs = years lived with disability; DALYs = disability-adjusted life years.

Despite its inherent complexity and challenges, metabolomics is evolving into a crucial tool for defining patient phenotypes concurrently with other -omics platforms (Figure 3). In this review, we have discussed the various metabolite types that have been reported to serve as biomarkers in infectious diseases, along with drugs developed for personalized treatment, diagnostic kits (POCTs) and companion diagnostics that have received FDA approval. Although metabolomics is still regarded to be in its infancy in the field of personalized medicine, it will inevitably take the lead in PM.
